# Transplantation of Human Pluripotent Stem Cell-Derived Cardiomyocytes for Cardiac Regenerative Therapy

**DOI:** 10.3389/fcvm.2021.707890

**Published:** 2021-11-08

**Authors:** Sophia E. Silver, Ryan W. Barrs, Ying Mei

**Affiliations:** ^1^Bioengineering Department, Clemson University, Clemson, SC, United States; ^2^Department of Regenerative Medicine and Cell Biology, Medical University of South Carolina, Charleston, SC, United States

**Keywords:** human pluripotent stem cell-derived cardiomyocytes, cardiovascular disease, cell therapy, regenerative medicine, tissue engineering

## Abstract

Cardiovascular disease is the leading cause of death worldwide and bears an immense economic burden. Late-stage heart failure often requires total heart transplantation; however, due to donor shortages and lifelong immunosuppression, alternative cardiac regenerative therapies are in high demand. Human pluripotent stem cells (hPSCs), including human embryonic and induced pluripotent stem cells, have emerged as a viable source of human cardiomyocytes for transplantation. Recent developments in several mammalian models of cardiac injury have provided strong evidence of the therapeutic potential of hPSC-derived cardiomyocytes (hPSC-CM), showing their ability to electromechanically integrate with host cardiac tissue and promote functional recovery. In this review, we will discuss recent developments in hPSC-CM differentiation and transplantation strategies for delivery to the heart. We will highlight the mechanisms through which hPSC-CMs contribute to heart repair, review major challenges in successful transplantation of hPSC-CMs, and present solutions that are being explored to address these limitations. We end with a discussion of the clinical use of hPSC-CMs, including hurdles to clinical translation, current clinical trials, and future perspectives on hPSC-CM transplantation.

## Introduction

Cardiovascular disease (CVD) is the leading cause of death worldwide (1). In the United States alone, CVD is responsible for ~655,000 deaths and contributes to $200 billion in spending each year (2). CVD can lead to myocardial infarction (MI), also known as a “heart attack,” which results in restricted blood flow and extensive cell death within the infarct zone. Due to the limited regenerative capacity of the human heart, infarcted myocardium is replaced by fibrotic scar tissue with inferior contractile performance. Over time, pathological remodeling leads to ventricular wall thinning, which can progress to heart failure (3). There is currently no treatment available that can restore lost cardiomyocytes after MI, and conventional therapies typically only manage the symptoms (3, 4). Heart transplantation is the only therapy capable of replacing a failing heart, but the shortage of viable donor organs and need for lifelong immunosuppression presents its own set of challenges for heart transplantation as a therapy (5). Therefore, alternative approaches that can restore the function of the patient's heart and replace infarcted myocardium would be a transformative development in cardiovascular medicine.

Stem cell therapy for cardiac regenerative medicine has drawn major interest due to the promising capacity of stem cells to differentiate into functional tissue. Several sources have been investigated for stem cell-mediated cardiac regenerative therapy, including both human adult stem cells and human pluripotent stem cells (hPSCs) (6). Unlike adult stem cells, hPSCs have a proven capacity to derive functional cardiomyocytes, and their scalable production *in vitro* has made hPSCs a favorable cell source for cardiac regenerative medicine (7, 8).

**Figure 1:**
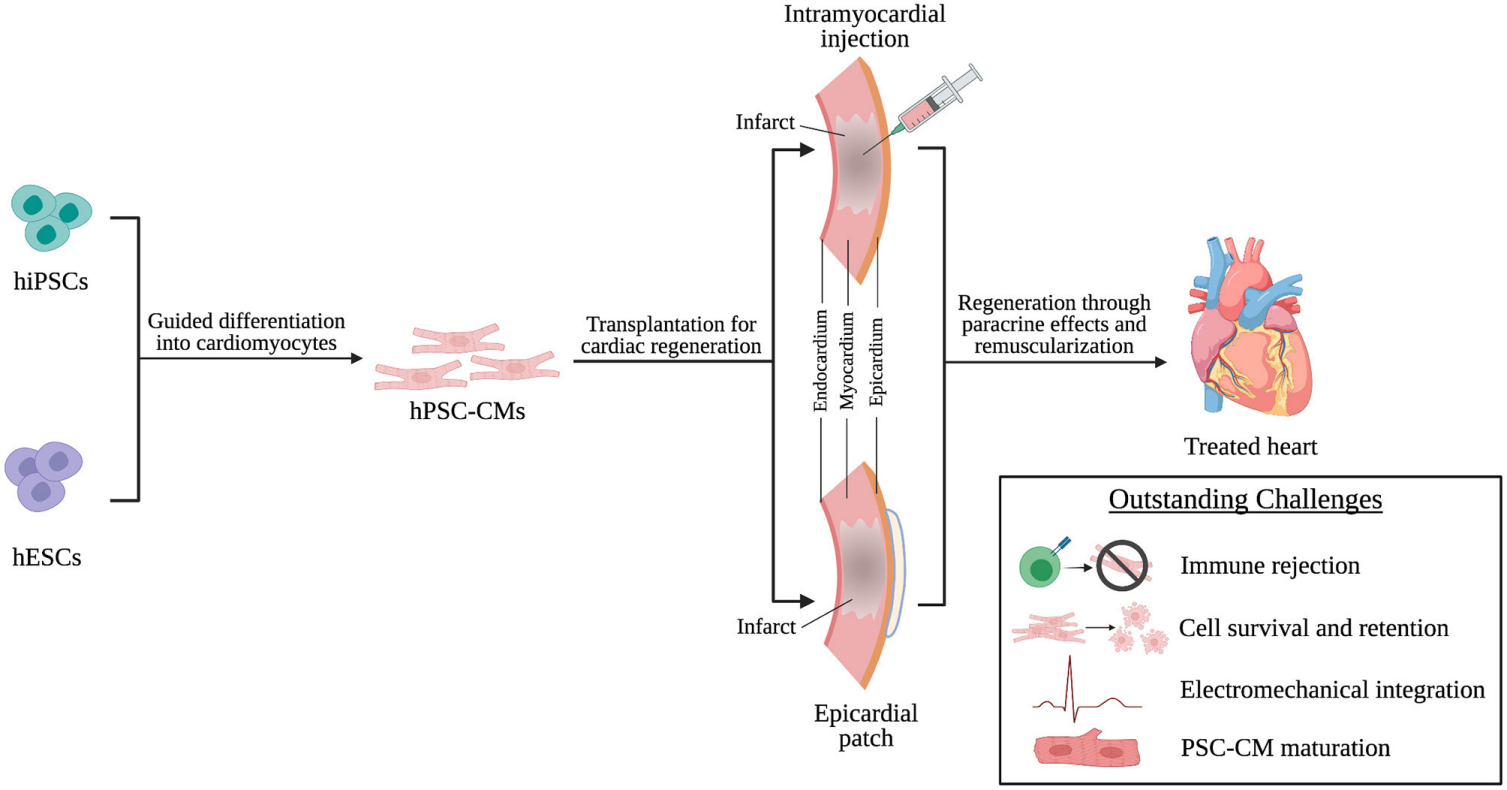
hPSC-CMs are differentiated from hiPSCs and hESCs and transplanted into the infarcted heart through intramyocardial injection or epicardial patches as a cardiac regenerative therapy. Following transplantation, regeneration is driven by paracrine effects of and remuscularization of myocardial tissue by engrafted hPSC-CMs. However, challenges persist that limit successful transplantation of hPSC-CMs and will need to be addressed to achieve effective clinical translation.

This review will discuss the origins and characteristics of human pluripotent stem cell-derived cardiomyocytes (hPSC-CMs) and how they are implemented in transplantation techniques (Figure 1). Additionally, we will discuss the potential mechanisms through which these transplantation strategies improve cardiac function and what challenges limit effective hPSC-CM transplantation. Finally, we will end with a discussion of challenges facing clinical translation of these transplantation strategies (Figure 1), current clinical trials involving hPSC-CMs, and future considerations in the field of transplantation of hPSC-CM for cardiac regenerative therapies.

## Human Pluripotent Stem Cell Sources and Differentiation into Cardiomyocytes

Human embryonic stem cells (hESCs) are a form of hPSCs isolated from human blastocysts cultured for *in vitro* fertilization. hESCs are capable of unlimited self-renewal and can differentiate into derivatives of all three germ layers (9). The differentiation potential of hESCs has been harnessed to reproducibly generate cardiomyocytes (hESC-CMs) (10). As the production of hESCs involves the destruction of human embryos, there are many ethical controversies that accompany the use of hESCs (11). To overcome these ethical concerns, human induced pluripotent stem cells (hiPSCs) have been explored as a cardiomyocyte source. hiPSCs are reprogrammed somatic cells with the capacity to differentiate into cells of all three embryonic germ layers. The concept behind the development of hiPSCs was that the genes that allow a cell to maintain its pluripotency could be overexpressed in a somatic cell and reprogram it to an ESC-like state (12). Viral vectors (12, 13) as well as recombinant proteins (14) and micro RNAs (15, 16) have been used to reprogram adult human cells to a pluripotent state.

The major methods to derive CMs from hPSCs are embryoid body differentiation, monolayer differentiation, and inductive differentiation (17). Common among all of these methods is the principle of mimicking endogenous embryonic cardiovascular development, including modulation of Wnt, Activin/Nodal, TGF-β, and BMP signaling pathways (18–21). Currently, hPSC-CM purity following differentiation can reach over 90% (18, 19, 21). The phenotype of hPSC-CMs resembles that of fetal CMs. For instance, they are morphologically small, spontaneously beat, lack T-tubules, and have underdeveloped and inefficient calcium handling (22). Developments in methods for differentiation and culture are working toward the goal of producing hPSC-CMs with a more mature phenotype, as will be discussed later in this review.

## Transplantation Strategies

Delivery routes for cardiac cell therapies have included intravenous injection, intramyocardial injection, intracoronary injection, intrapericardial transplantation, and epicardial patches. Each of these methods have their own strengths and weaknesses regarding cell retention and functional outcomes (23). For hPSC-CM transplantation, intramyocardial injection and epicardial patches have been the most popular delivery routes in pre-clinical studies and first-in-human clinical trials. Therefore, we will focus on these two transplantation strategies in this review.

### Intramyocardial Injection

Early studies in the transplantation of hPSC-CM involved intramyocardial injection of single cell suspensions in mouse (24), rat (25), guinea pig (26), and swine models (27). Although hPSC-CMs demonstrated the ability of to partially remuscularize the animal hearts, cell retention and survival rates were low, and there was insufficient evidence of functional integration. To improve hPSC-CM survival post-transplantation, Murry et al. developed a pro-survival cocktail that led to enhanced cell survival after transplantation, robust cardiac remuscularization, and functional improvement in both small (28–32) and large (33) animal models of ischemic injury. Murry's group later showed that hPSC-CM injection in a non-human primate model of MI results in extensive remuscularization and electromechanical coupling of grafted cells to host myocardium (33, 34). They further confirmed the ability of the engrafted hPSC-CMs to restore function in the non-human primate heart by demonstrating improved left ventricular ejection fraction. However, they also observed transient graft-associated ventricular arrhythmias, which was attributed to the ectopic pacemaker activity of the engrafted hPSC-CMs (33, 34).

To aid in cell retention following engraftment of hPSC-CM, recent studies have explored injectable three-dimensional hPSC-CM microtissues to provide critical cell-cell interactions and reduce anoikis. For example, Moon et al. demonstrated reduced fibrosis, improved fractional shortening, and prolonged survival of 5–10 cell hPSC-CM aggregates injected into infarcted rat hearts (35). Larger scale hPSC-CM spheroids containing 200,000 cells each have also been implemented to promote improvement in fractional shortening and engraftment rates following infarction in a murine model (36). Spheroids consisting of hPSC-CMs have also been implanted into a porcine model of heart failure, leading to functional improvement (37). However, graft-associated arrhythmias were observed in the swine transplanted with hiPSC-CM spheroids.

### Epicardial Patches

Epicardial patches refer to engineered heart tissues that are attached to the outer surface of the heart, usually adjacent to the infarct region. In addition to providing mechanical support, epicardial patches function as a scaffold to provide cell-ECM interactions that promote hPSC-CM survival and engraftment post-transplantation as well as secretion of cardioprotective paracrine factors (38, 39). For example, rodent models of chronic ischemia have been treated with epicardial patches and demonstrated long-term retention of grafts (40). Despite this progress, patches are not immediately perfused post-transplantation and can be isolated from host myocardium by a layer of fibrotic tissue, limiting nutrient diffusion to cells within the construct post-transplantation (40). To address this, porous patches seeded with hPSC-CMs have been investigated to examine whether the porous nature of the patch would allow sufficient nutrient and oxygen exchange to engrafted cardiomyocytes (41). Munarin et al. have also recently demonstrated that the incorporation of alginate microspheres containing angiogenic factors in hPSC-CM scaffolds could lead to enhanced host vasculature infiltration into the scaffolds and improved cell survival when implanted in a rodent model of acute MI (42).

To improve vascular integration with host myocardium, vascular cells (i.e., endothelial cells) have been incorporated into epicardial patches with hPSC-CMs. Biodegradable scaffolds seeded with a triculture of hPSC-CMs, human umbilical vein endothelial cells (HUVECs), and embryonic fibroblasts promoted graft vascularization and anastomosis with host coronary vasculature in rodent hearts (43). Ye et al. combined the use of biomaterials and multiple cell types to investigate a 3D fibrin patch loaded with the pro-survival factor insulin-like growth factor-1 (IGF-1)-encapsulated microspheres seeded with hPSC-CMs, endothelial cells (ECs), and smooth muscle cells (SMCs). When implanted in a porcine model of acute MI, all three cell types integrated with the host, and physiological improvements were observed in terms of improved left ventricle function, myocardial metabolism, and ventricular wall stress (44). Advances in engineered heart tissue have led to the fabrication of clinical scale human cardiac muscle patches (hCMP) consisting of 3D fibrin scaffolds seeded with hPSC-CMs, -ECs, and -SMCs (45, 46). The hCMPs exhibited 10% engraftment at 4 weeks post-implantation and promoted significant improvement in cardiac function and reduction in wall stress and infarct size (46).

Scaffold-free approaches have also been used to create epicardial patches. Cell sheet technology, developed by Okano et al., involves coating culture dishes with PNIPAAm, a thermo-responsive polymer, to release cells and produce cell sheets upon changing temperature (47). This technique was recently used to fabricate cardiac tissue sheets from hPSCs, which were then implanted into small and large animal injury models to demonstrate their therapeutic potential (48–51). In addition, Murry et al. developed pre-vascularized cell sheets with enhanced survival and anastomosis with host vasculature upon transplantation in healthy rodent hearts (52).

## Mechanisms of Improving Cardiac Function

### Remuscularization

A major goal of cardiac regenerative medicine is to remuscularize the infarcted myocardium, restoring the muscle that was lost to ischemic injury (53). Intramyocardial injection of hPSC-CMs allows the engrafted hPSC-CMs to integrate with host myocardium and directly contribute to contractile function. Functional integration has been evidenced by the formation of gap junctions between host and engrafted cardiomyocytes in various small (28, 29, 39) and large (33, 34) animal models.

Epicardial patches can improve hPSC-CM engraftment, provide partial remuscularization to infarcted myocardium, and augment left ventricular function in a dose-dependent manner (54). However, the fibrotic tissue between the patch and myocardium can reduce long-term survival of the hPSC-CMs and prohibit the formation of electromechanical junctions between the engrafted hPSC-CMs and host myocardium, leading to unsynchronized contractions (29).

### Paracrine Effects

In many instances of intramyocardial hPSC-CM transplantation, functional recovery has occurred even without significant hPSC-CM engraftment, leading researchers to hypothesize that paracrine factors (e.g., cytokines, extracellular vesicles, etc.) released by the transplanted cells are partially responsible for improvements in damaged myocardium. This concept was explored through single-cell profiling of hPSC-CMs following their transplantation in a murine acute MI model. Left ventricular function was improved despite limited engraftment, and hPSC-CMs were found to release high levels of proangiogenic and anti-apoptotic factors, suggesting functional benefits came from paracrine activity (55). This is further supported by similar functional recovery obtained by injection of hPSC-cardiac cells and hPSC-cardiac cell-secreted exosomes into infarcted porcine hearts (56). Cardioprotective microRNAs have been identified in hiPSC-CM-derived extracellular vesicles, and extended delivery *via* a hydrogel patch improved cardiac recovery (57).

Due to subnormal formation of electromechanical junctions with host myocardium, epicardial patches typically repair injured hearts through mechanical support and paracrine effects. Given the fibrotic separation, vascular integration between epicardial patches and host myocardium may play a critical role in transporting patch-derived paracrine factors into myocardium (39, 41).

## Challenges to Improve hPSC-CM Transplantation Strategies

Although progress has been made in the field of hPSC-CM transplantation, challenges still face transplantation and clinical translation of hPSC-CM therapies. In the next two chapters, we will first discuss the challenges that face the development of successful hPSC-CM transplantation techniques and then the outstanding challenges that limit safe and effective clinical translation of these techniques.

### Immune Rejection

Transplantation of allogenic cells or tissues can elicit an immune response that ultimately leads to graft rejection and can have harmful consequences for the transplant recipient. Solutions include major histocompatibility (MHC)-matching and the production of hPSC banks (58). Shiba et al. performed an MHC matching study in which they transplanted allogenic non-human primate PSC-CMs 14 days after injury in a cynomolgus monkey model of MI. They observed improved cardiac function, along with electrical coupling with the host myocardium and no evidence of immune rejection in the MHC-matched PSC-CM group, suggesting the safety of transplanting MHC-matched, donor-derived hPSC-CMs in humans (59). Eventually, autologous transplantation of hPSC-CMs would be ideal and hiPSC-CMs, in particular, offer a promising source of patient-derived cells. However, manufacturing challenges must be overcome to make autologous hPSC-CM transplantation practical for clinical use.

### Cell Survival and Retention

Low cell survival and retention after transplantation is a central obstacle in the development of effective hPSC-CM-based cardiac regenerative therapy (60, 61). To improve survival of intramyocardial injected hPSC-CM single cells, a pro-survival cocktail for injection was developed to address common causes of graft death (62). A recent study found that co-transplantation of hiPSC-CMs with ready-made microvessels from adipose tissue resulted in a six-fold improvement in hiPSC-CM cell survival (63). To promote cell survival in epicardial patches, pre-vascularization strategies have been explored to promote anastomosis of the patches with host vasculature (64, 65). Going forward, novel bioengineering approaches (e.g., biomaterials and cellular engineering) could improve hPSC-CM retention (23).

### Electromechanical Integration of the Graft

Due to the wound healing response following MI and intramyocardial injections, fibrosis develops around transplanted hPSC-CMs. This affects signal propagation and proper electromechanical integration of the graft, leading to arrhythmias (66). In studies of hPSC-CM transplantation, intramyocardial engraftment into non-human primates (33, 59) and porcine models (67) was associated with transient ventricular arrhythmias (68). To solve these issues, conductive scaffolds can be used to aid in signal propagation (66). Furthermore, engrafted hPSC-CMs have an immature phenotype associated with spontaneous beating, which will affect the electrical signaling in the heart (68). To decrease the presence of arrhythmias, hPSC-CM maturation and ventricular subtype-specific differentiation protocols would be useful to eliminate pacemaker-like activity from engrafted cells (22, 34). Epicardial transplantation of hPSC-CM patches has not been shown to elicit arrhythmias in guinea pig (69) and porcine (46) hearts. However, this could be due to fibrotic isolation of the graft and lack of electromechanical coupling with host myocardium (70).

### hPSC-CM Maturation

As mentioned, hPSC-CM have an immature phenotype. Maturation of hPSC-CM involves physiological hypertrophy associated with organization of sarcomeric structure, along with presence of T-tubules (71). hPSC-CM maturation also involves more efficient calcium handling, improved electrophysiological properties and higher contractile force (72). Therefore, transplanted CM with properties that more closely resemble adult myocardium would reduce the risk of arrhythmias and have improved contractile properties (73). Several methods have been investigated for maturation of hPSC-CM, including long-term culture, changes in the culture substrate stiffness, electrical stimulation, and biochemical cues (73). Mechanical loading has also been used to stimulate maturation in iPSC-derived cardiac tissue (74, 75). Additionally, tissue engineering methods have been employed to promote maturation. Engineered heart tissue made from a co-culture of hESC-CM and hESC-derived epicardium promoted hESC-CM maturation in terms of enhanced contractility, myofibril structure, and calcium handling (76). Electrical training of hPSC-CMs in three-dimensional culture system has also contributed to advanced morphological maturation of hiPSCs (77). Three-dimensional culture containing multiple cell types has also been shown to promote a more mature phenotype of hiPSC-CMs (78, 79).

## Clinical ApplicationS of hPSC-CMs

### Challenges in Clinical Translation of hPSC-CMs

There are several safety concerns in the clinical use of hPSC-CM treatments. In addition to potential tumorigenicity and immune rejection, a major roadblock for intramyocardial injection is hPSC-CM graft-associated arrhythmias. Recent evidence has demonstrated the feasibility of pharmacological therapy for hPSC-CM-induced arrhythmias after intramyocardial injection (80). Arrhythmia risk may increase with graft size and, therefore, thorough cell dose-response studies are needed. While studies with hPSC-CM epicardial patches have mostly indicated no arrhythmic burden, the long-term effects of their subnormal electromechanical integration are unclear (81).

Most cardiac injury models to date can be classified as acute or subacute MI, with transplantation occurring within minutes to days after infarction. In a clinical setting, hPSC-CM therapy would often be performed months to years after MI as a last resort in patients with chronic heart failure (68). While hPSC-CM transplantation at 2 weeks post-MI can improve cardiac function in rats (82), transplantation at 1 month post-MI showed no functional benefit in rats (83) or guinea pigs (84). Sawa et al. showed that hPSC-CM cell sheet transplantation 1 month post-MI can improve cardiac function in swine, but there was no evidence of graft-host electromechanical integration and very few cells survived long-term (50), which could be attributed to the established fibrotic environment in chronic MI. These discrepancies necessitate further evaluation of animal models of chronic heart failure to determine the potential of hPSC-CM transplantation in a more clinically applicable setting.

Scalable manufacturing of clinical-grade hPSC-CMs is also a serious challenge for clinical use and, therefore, several recent studies have focused on large-scale production of clinical-grade hPSC-CMs. Master iPSC cell banks have been developed for clinically compliant sourcing of PSC-derived cells under current good manufacturing practice (cGMP) (85). To increase cell production, PSC aggregate culture and differentiation systems that produce 10^9^ hPSC-CMs in a 1 L flask have been developed (86). Serum-free (87) and human serum-based (54) construction protocols for engineered heart tissue (EHT) patches have also been developed to adapt to cGMP for clinical applications.

Finally, there is a lack of consensus on the characterization and assessment of hPSC-CM differentiation and maturity (i.e., cell surface markers). Consistency in the assessment of hPSC-CM products is necessary to ensure their quality, reproducibility, and safety for use in humans. To this end, an unbiased integrative proteomics approach could offer comprehensive assessment of hPSC-cardiomyocyte maturation (88).

### First In-human Clinical Trials With hPSC-CMs

Despite the outstanding challenges in the field, first-in-human clinical trials have recently begun involving the transplantation of hPSC-CMs (Table 1). The first use of hPSC-CMs in humans took place in 2019 in Nanjing, China, and involved intramyocardial injection of hiPSC-CMs in patients with chronic ischemic cardiomyopathy (89). However, cell injection occurred alongside coronary artery bypass grafting, limiting the ability to delineate the therapeutic benefits of hiPSC-CM transplantation. In Japan, a trial at Osaka University is exploring transplantation of an allogeneic hiPSC-CM cell sheet as a sole therapy for ischemic cardiomyopathy (90). Heartseed Inc., a Japan-based biotechnology company led by Prof. Keiichi Fukuda, recently gained approval for a Phase I/II clinical trial of intramyocardial injection of three-dimensional hiPSC-CM spheres to treat heart failure. The largest trial to date has been registered in Germany at University Medical Center Goettingen, investigating the remuscularization capacity of engineered heart tissue containing hiPSC-CMs and stromal cells in patients with heart failure with reduced ejection fraction (HFrEF).

**Table 1:** Current clinical trials involving hPSC-CM transplantation for heart repair.

**Trial ID**	**Sponsor**	**Title**	**Condition**	**Intervention**	**Estimated enrollment**	**Start date**	**Country**
NCT03763136	Help therapeutics	The study of human epicardial injection with allogenic induced pluripotent stem cell-derived cardiomyocytes in ischemic heart failure	Heart failure	Intramyocardial injection of allogenic hiPSC-CMs at time of coronary artery bypass grafting surgery	5	May 2019	China
jRCT2053190081	Osaka University Hospital	Clinical trial of human (allogeneic) iPS cell-derived cardiomyocytes sheet for ischemic cardiomyopathy	Ischemic cardiomyopathy	Human (allogeneic) iPS cell derived-cardiomyocyte sheet transplantation	10	January 2020	Japan
NCT04396899	University Medical Center Goettingen	Safety and efficacy of induced pluripotent stem cell-derived engineered human myocardium as biological ventricular assist tissue in terminal heart failure	Heart failure	Implantation of EHM on dysfunctional left or right ventricular myocardium in patients with HFrEF (EF <35%).	53	February 2020	Germany
jRCTa032200189	Heartseed Inc.	Safety study of regenerative therapy with allogeneic induced pluripotent stem cell-derived cardiac spheres for severe heart failure (Regenerative cardiac spheres)	Severe heart failure patients with NYHA class III or higher (HFrEF by Dilated Cardiomyopathy)	Intramyocardial injection of 5 × 10^7^ iPSC-derived cardiomyocytes by open-heart surgery	3	November 2020	Japan

## Conclusions and Future Considerations

Transplantation of hPSC-CMs has proven to be a viable strategy for cardiac regenerative therapies. Single-cell injection and tissue-level engineered constructs have served as the basis for promoting functional improvements in injured myocardium. Future research needs to focus on addressing the limitations currently facing the field, as discussed in this review. In particular, the development of a viable strategy to prevent graft-associated arrythmia will have immediate clinical impacts for intramyocardial injection of hPSC-CMs. In addition, paracrine factors play a central role in hPSC-CM mediated functional recoveries; therefore, developing methods to enhance hPSC-CM cardioprotective secretome would have significant impacts to the field. Lastly, optimal doses of PSC-CMs for heart repair need to be determined for safe and effective application in humans.

In summary, although the clinical translation of hPSC-CM transplantation faces several significant limitations, immense progress has been made in recent years in the development of potential strategies for hPSC-CM regenerative therapies. It has been proven that engrafted hPSC-CM can make meaningful connections with host cardiomyocytes and provide paracrine factors that stimulate functional recovery of host myocardium. Furthermore, strategies for producing cells at a clinical scale have been explored, as well as methods to mitigate immune rejection, reduce incidence of cardiac arrhythmias, and mature hPSC-CMs.

## Author Contributions

SS was responsible for manuscript development and primary authorship under the guidance of YM. RB contributed to organization and conceptualization of the work and was lead author on section First In-Human Clinical Trials With hPSC-CMs. YM contributed to article organization and conceptualization. All authors contributed to the article and approved the submitted version.

## Funding

This work was supported by the National Institutes of Health (1F31 HL156541, 1R01HL133308, 8P20 GM103444), and the National Science Foundation (NSF—EPS-0903795, NSF1655740).

## Conflict of Interest

The authors declare that the research was conducted in the absence of any commercial or financial relationships that could be construed as a potential conflict of interest.

## Publisher's Note

All claims expressed in this article are solely those of the authors and do not necessarily represent those of their affiliated organizations, or those of the publisher, the editors and the reviewers. Any product that may be evaluated in this article, or claim that may be made by its manufacturer, is not guaranteed or endorsed by the publisher.
